# Safety and Clinical Efficacy of Yangxue Qingnao Granules in the Treatment of Chronic Cerebral Circulation Insufficiency: A Systematic Review and Meta-Analysis

**DOI:** 10.1155/2019/8484263

**Published:** 2019-08-14

**Authors:** Jianbo Guo, Xingjiang Xiong, Yu Cao, Yukun Ding, Qingyong He

**Affiliations:** ^1^Department of Cardiology, Guang'anmen Hospital, China Academy of Chinese Medical Sciences, Beijing 100053, China; ^2^Beijing University of Chinese Medicine, Beijing 100029, China; ^3^Xiyuan Hospital, China Academy of Chinese Medical Sciences, Beijing 100091, China

## Abstract

To evaluate the safety and clinical efficacy of Yangxue Qingnao (YXQN) granules in the treatment of chronic cerebral circulation insufficiency (CCCI), electronic databases—PubMed, Embase, CNKI, VIP, and Wangfang—were searched for randomized controlled trials (RCTs) published up to January 2019. GRADE and RevMan 5.3.0 were used for grading and analysis, respectively. Fifteen trials involving 1211 CCCI patients were included. Subgroup analysis was performed owing to study heterogeneity. Compared to nimodipine plus routine treatment, YXQN granules plus routine treatment were more effective in increasing basilar artery (BA) blood flow velocity (mean difference (MD) = 3.34, 95% confidence interval (CI) = [2.31, 4.37], *P* < 0.00001), vertebral artery (VA) blood flow velocity (MD = 0.52, 95% CI = [0.27, 0.76], *P* < 0.0001), and internal carotid artery (ICA) blood flow velocity (MD = 7.46, 95% CI = [2.01, 12.90], *P*=0.007). In improving the clinical efficacy of traditional Chinese medicine (TCM) for symptoms such as headache, dizziness, and insomnia, YXQN granules plus routine treatment were shown to be superior to the following control treatments: nimodipine plus routine treatment (mean difference (M-H) = 4.21, 95% CI = [2.49, 7.12], *P* < 0.00001), flunarizine plus routine treatment (mean difference (M-H) = 3.92, 95% CI = [1.36, 11.29], *P*=0.01), troxerutin plus routine treatment (mean difference (M-H) = 4.79, 95% CI = [2.20, 10.42], *P* < 0.00001), and routine treatment (mean difference (M-H) = 6.13, 95% CI = [1.48, 25.34], *P*=0.01). Risk of bias was assessed in 15 trials. One analysis was graded using GRADE and showed poor results. Adverse events were not reported explicitly in all but one trial. Thus, this meta-analysis suggests that YXQN granules may be beneficial for patients with CCCI. However, owing to the poor quality of the clinical trials and small sample sizes, a definite conclusion on the efficacy and safety of YXQN granules cannot be drawn from existing information.

## 1. Introduction

Chronic cerebral circulation insufficiency (CCCI) is a common and frequently occurring ischemic cerebrovascular disease, with a complex etiology and recurrent symptoms. According to a recent report, two-thirds of the over-65-year-old population in China suffer from CCCI [1]. It is mostly caused by hypertension, arteriosclerosis, hyperlipidemia, and diabetes [1–4]. In the 1990s, the Ministry of Health and Welfare of Japan commissioned the “Research Class on the Definition and Diagnostic Criteria of Cerebral Arteriosclerosis Diseases” to study the condition and proposed that the name should be “chronic cerebral circulation insufficiency” [5].

Western medicine treats CCCI by interventions targeting the risk factors (including hypertension, hyperlipidemia, diabetes, and smoking), etiology (cranial artery stenosis, cerebrovascular lesions, and leukoporosis), cerebral microcirculatory perfusion (using betahistine, flunarizine, and nimodipine), and cognitive deficits (with agents such as cytidine diphosphate choline, compound cerebral peptide ganglioside injection, and compound triglyceride injection) [6, 7]. Because of the complexity of CCCI, there is a lack of effective drugs in modern medical treatment. Surgical therapy has made some progress in recent years; nevertheless, this treatment is not easily accepted by patients because of the risk of adverse events and further complications after the operation [8–11]. Thus, there is a need for other effective treatments for CCCI.

Yangxue Qingnao (YXQN) granules are a traditional Chinese medicine (TCM) mainly composed of Angelicae Sinensis Radix, Chuanxiong Rhizoma, Paeoniae Radix Alba, Rehmanniae Radix Praeparata, Uncariae Ramulus Cum Uncis, Spatholob Caulis, Prunellae Spica, Cassiae Semen, Margaritifera Concha, Corydalis Rhizoma, and Asari Radix et Rhizoma. According to TCM theory, YXQN granules function to activate blood circulation and remove blood stasis, nourish the blood and liver, and dredge channels and collaterals [12, 13]. YXQN granules are believed to be capable of treating chronic cerebral insufficiency by nourishing the liver and blood, promoting blood circulation, and removing blood stasis, thereby improving the clinical symptoms of such patients. Although the pharmacological mechanism of YXQN granules is not yet understood, its clinical application is relatively extensive [14–16]. The purpose of this systematic review and meta-analysis was to compare the efficacy and safety of YXQN granules with those of other drugs used to treat CCCI by reviewing clinical studies.

## 2. Materials and Methods

### 2.1. Search Strategies

This meta-analysis of clinical trials was conducted in accordance with the guidelines [17, 18] set out in the PRISMA Statement (PRISMA Checklist S1) and was registered on PROSPERO (CRD42019121368). The study retrieved all articles published before January 2019 by searching five electronic databases: the PubMed Database, the Chinese Science and Technology Periodical Database (Embase), the Chinese National Knowledge Infrastructure (CNKI) Database, the China Scientific Journal Database (VIP), and the Wangfang Database. The search words and MeSH terms used were “chronic cerebrovascular insufficiency” or “chronic cerebral circulation insufficiency” or “cerebral circulation insufficiency” or “cerebrovascular insufficiency” or “CCCI” and “Yangxue qingnao” or “yangxueqingnao” or “YXQN” or “Yang-Xue-Qing-Nao” and “random” and “groups” and “trial.”

### 2.2. Eligibility Criteria

All randomized controlled trials (RCTs) on the use of YXQN granules for CCCI met the inclusion criteria. All patients diagnosed with CCCI were considered for this review. We followed the diagnostic criteria for CCCI issued by the 16th Stroke Society of Japan in 2000: (1) dizziness, headache, and other conscious symptoms; (2) hypertension, changes in fundus arteriosclerosis, or vascular murmurs of the cerebral perfusion arteries; (3) no focal neurological signs in the brain; (4) no vascular organic encephalopathy on computed tomography (CT) or magnetic resonance imaging (MRI) of the head; (5) exclusion of other conditions that could cause the above symptoms; (6) the cerebral circulation tester showing decreased cerebral blood flow; and (7) digital subtraction angiography (DSA) or transcranial Doppler ultrasound (TCD) suggesting obstruction or stenosis of cerebral perfusion flow arteries, with criteria (1) to (4) being necessary conditions. The studies had to compare the curative effect of YXQN granules or routine treatment plus YXQN granules with routine treatment alone or with other medicines for CCCI.

The dosage of YXQN granules is 4 g as an undivided dose, taken three times a day. The main outcome measures were (1) basilar artery (BA) blood flow velocity, (2) vertebral artery (VA) blood flow velocity, and (3) internal carotid artery (ICA) blood flow velocity. Secondary outcome measures were the clinical efficacy of TCM symptoms, including the main symptoms of headache, dizziness, and insomnia.

### 2.3. Data Extraction and Analysis

Two authors (JG and XX) independently extracted data from each trial. Discrepancies were resolved through discussion, and the whole process was supervised by a third author. The following information was extracted from each trial: name of the researcher, the date of the trial, the total number of patients included, the proportion of men and women included, age distribution, grouping methods, intervention measures, outcome indicators, data statistics, and adverse events.

### 2.4. Risk-of-Bias Assessment

The two authors JG and XX judged the risk of bias based on the risk-of-bias assessment tool [19] and independently evaluated the methodological quality of the included RCTs using RevMan 5.3.0. Random sequence generation, allocation hiding, inconsistency of results, and selective result reporting were evaluated. Other biases included sample size, baseline characteristics, and inclusion and exclusion criteria. The quality of each included RCT was classified as low, unclear, or high risk of bias. Discrepancies were resolved by discussion with a third author (QH). If conditions were identical or similar, and data were sufficiently consistent, meta-analysis would be performed. Dichotomous data were analyzed using risk ratios (RRs) and continuous variables using mean differences (MDs) with 95% confidence intervals (CIs).

Heterogeneity between studies was evaluated and quantified by Cochran's *Q* test and Higgins' *I* ^2^ statistic. Data with low heterogeneity (*P* > 0.10 and *I* ^2^ < 50%) were assessed with a fixed-effects model; alternatively, data with high heterogeneity (*P* > 0.10 and *I* ^2^ > 50%) were assessed with a random-effects model. If significant interstudy heterogeneity was detected, further subgroup analysis was conducted based on possible clinical causes.

## 3. Results

### 3.1. Trial Search and Selection

**Figure 1:**
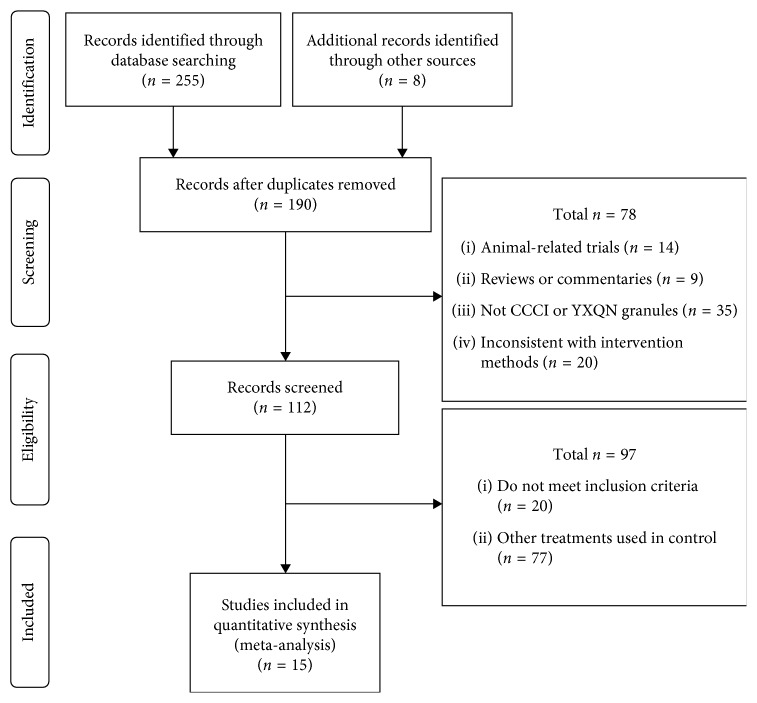
Selection process of this systematic review.

Two hundred fifty-five studies on the use of YXQN granules for CCCI, published before January 2019, were preliminarily retrieved from the five electronic databases. Eight studies were additionally identified through other sources. After deleting duplicate studies, 20 remaining English studies and 170 Chinese studies were selected for further examination. By reviewing the title and abstract, 14 animal trials, 9 reviews or commentaries, and 55 studies that did not meet the inclusion requirements for disease and treatment were excluded. Finally, a full-text review was conducted on the remaining 112 studies, according to the inclusion criteria of intervention, control, and outcomes. Of these, 97 were excluded and 15 studies [20–33] were included. The screening process is summarized in the study flowchart (Figure 1).

### 3.2. Trial Characteristics

Table 1 lists the characteristics of the included RCTs (*n*=15). Between 2003 and 2017, 15 suitable RCTs, involving 1211 participants aged 35 to 90 years, were published. The sample size per study ranged from 52 to 104 participants, with an average of 80.73 participants per trial. All studies stated that the included patients met the diagnostic criteria for CCCI. Five [16, 21, 25, 30, 33] studies clearly indicated that patients met the diagnostic criteria of the Japanese Ministry of Health and Welfare for CCCI. In the remaining 10 studies [20, 22–31], the inclusion criteria were determined by reading the full text. All 15 studies had two test arms. All the control groups were treated with YXQN granules plus routine therapy, which were compared with routine treatment alone in three studies [26, 28, 33], with flunarizine plus routine treatment in two studies [20, 24], with nimodipine tablets plus routine treatment in six studies [16, 21, 25, 30–32], with troxerutin plus routine treatment in three studies [22, 29, 31], and with cinnarizine tablets plus routine treatment in one study [23].

In all studies, YXQN was taken three times a day; the dose was 4 mg each time in one trial [23] and 4 g each time in the remaining studies. The course of the treatment ranged from 2 to 13 weeks.

All trials reported clinical efficacy, and seven trials reported TCD results. Ten trials reported adverse events, of which one reported the results in detail.

### 3.3. Methodological Quality

All included studies were RCTs, but only two studies [16, 31] (2/15, 13.33%) reported sequence generation methods, using the random number method and randomized table method, respectively. None of the studies described allocation concealment and blinding.

**Figure 2:**
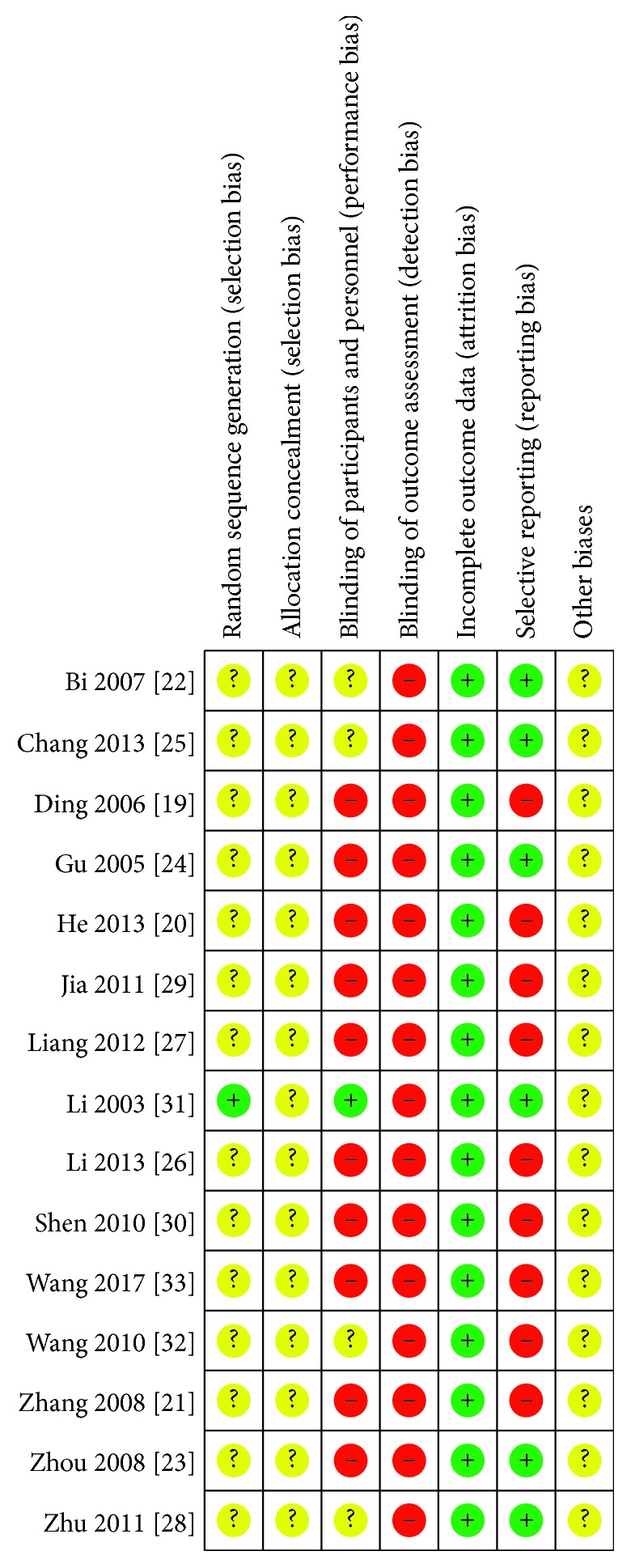
Risk-of-bias summary.

**Figure 3:**
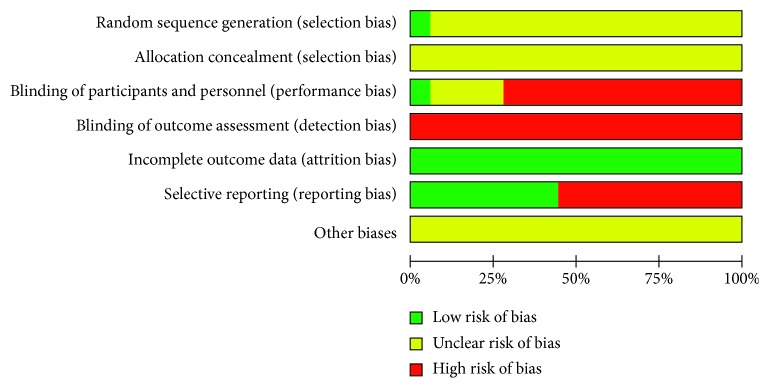
Risk-of-bias graph.

We were unable to interview the researchers from the 15 studies and only used the methods reported in the study to determine the risk of bias. All 15 studies that reported all outcomes from those mentioned in Materials and Methods (15/15, 100%) were assessed as having a low risk. In assessing the risk of selective reporting, six studies [16, 23–25, 28, 31] with selective reporting were assessed as having a low risk (6/15, 40%), and the remaining nine studies were assessed as having a high risk because the results were not well described (9/15, 60%). No trial provided estimates of sample size determined before the study commenced, and all trials were assessed as having a high or uncertain risk of bias in this section (Figures 2 and 3). The GRADE summary of five randomized controlled trials comparing YXQN granules plus routine treatment with nimodipine tablets plus routine treatment in CCCI is shown in Table 2.

### 3.4. BA Blood Flow Velocity

**Figure 4:**
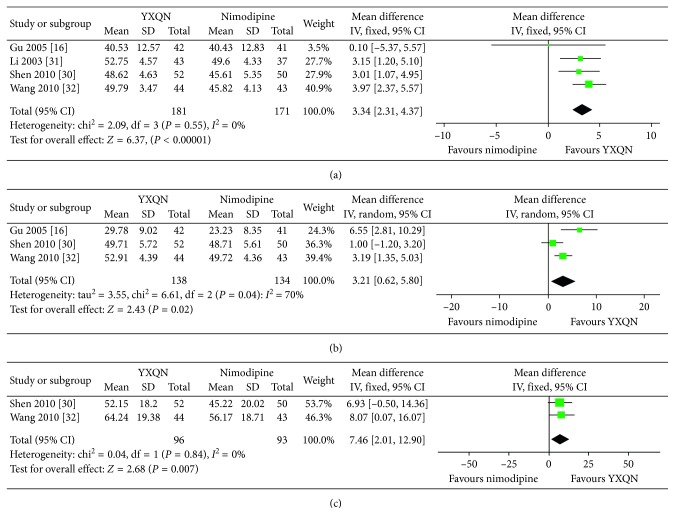
Forest plot of the comparisons between YXQN granules plus routine treatment and nimodipine tablets plus routine treatment. (a) Comparison for the outcome of the BA blood flow velocity. (b) Comparison for the outcome of the VA blood flow velocity. (c) Comparison for the outcome of the ICA blood flow velocity.

BA blood flow velocity was used to measure outcomes in four experiments. There was no significant difference in the BA blood flow velocity between the experimental group and the control group before treatment. The BA blood flow velocity was compared after treatment with YXQN granules plus routine treatment and treatment with nimodipine tablets plus routine treatment [16, 30–32]. Comprehensive analysis of the four studies showed that BA blood flow velocity after treatment with YXQN granules plus routine treatment was higher than that after treatment with nimodipine tablets plus routine treatment. Meta-analysis showed significant differences between the two groups (MD: 3.34, 95% CI: 2.31–4.37, *P* < 0.00001, *I* ^2^ = 0%) (Figure 4(a)).

### 3.5. VA Blood Flow Velocity

VA blood flow velocity was used to assess outcomes in three experiments. There was no significant difference in VA blood flow velocity between the experimental group and the control group before treatment. VA blood flow velocity after treatment was compared for the group receiving YXQN granules plus routine treatment and that receiving nimodipine tablets plus routine treatment [16, 30, 32]. Three studies, involving 272 patients, showed heterogeneity (*I* ^2^ = 70%); thus, a random-effects model was used. Meta-analysis results showed significant differences between the two groups (MD: 3.21, 95% CI: 0.62–5.80, *P*=0.02, *I* ^2^ = 70%) (Figure 4(b)).

### 3.6. ICA Blood Flow Velocity

ICA blood flow velocity was used to assess outcomes in two experiments. There was no significant difference in ICA blood flow velocity between the experimental group and the control group before treatment. ICA blood flow velocity after treatment was compared for the group receiving YXQN granules plus routine treatment and that receiving nimodipine tablets plus routine treatment [30, 32]. Comprehensive analysis of the two studies showed that ICA blood flow velocity after treatment with YXQN granules plus routine treatment was higher than that after treatment with nimodipine tablets plus routine treatment. Meta-analysis results showed significant differences between the two groups (MD: 7.46, 95% CI: 2.01–12.90, *P*=0.007, *I* ^2^ = 0%) (Figure 4(c)).

### 3.7. Clinical Efficacy of TCM Symptoms

**Figure 5:**
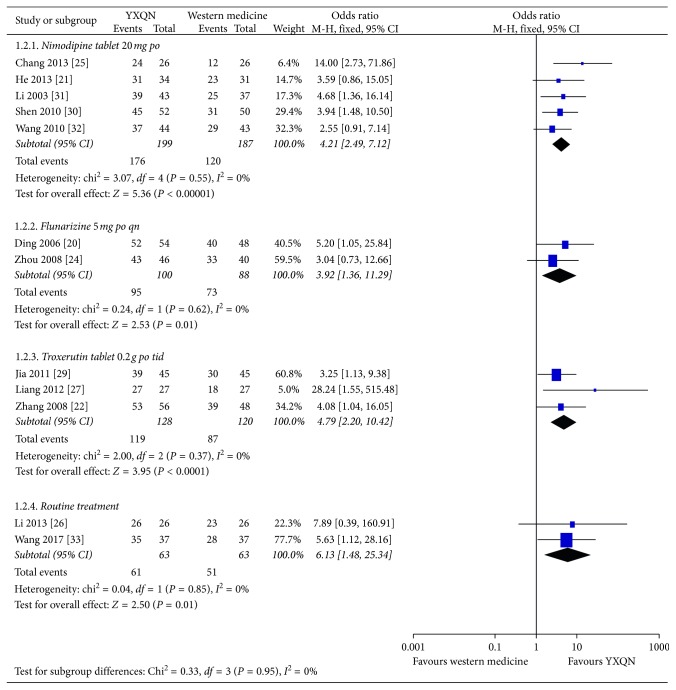
Forest plot of subgroup analysis of the clinical efficacy of TCM syndromes.

Fourteen trials studied the clinical efficacy of TCM syndromes as a result index. Two trials [20, 25] found that YXQN granules plus routine treatment were superior to compound *Salvia miltiorrhiza* tablets plus routine treatment in terms of the clinical efficacy of TCM syndromes (M-H: 2.36, 95% CI: 1.14–4.90, *P*=0.02, *I* ^2^ = 0%). Five trials [21, 25, 30–32] also found that YXQN granules plus routine treatment were superior to nimodipine tablets and routine treatment in terms of the clinical efficacy of TCM syndromes (M-H: 4.21, 95% CI: 2.49–7.12, *P* < 0.00001, *I* ^2^ = 0%). Two trials [20, 24] further found that YXQN granules plus routine treatment were superior to flunarizine plus routine treatment in the clinical efficacy of TCM syndromes (M-H: 3.92, 95% CI: 1.36–11.29, *P*=0.01, *I* ^2^ = 0%). Additionally, three trials [22, 27, 29] found that YXQN granules plus routine treatment were superior to troxerutin plus routine treatment in the clinical efficacy of TCM syndromes (M-H: 4.79, 95% CI: 2.20–10.42, *P* < 0.0001, *I* ^2^ = 0%). Moreover, two trials [26, 33] found that YXQN granules plus routine treatment were superior to conventional treatment in the clinical efficacy of TCM syndromes (M-H: 6.13, 95% CI: 1.48–25.34, *P*=0.01, *I* ^2^ = 0%) (Figure 5).

### 3.8. Adverse Effects

Ten trials [20–26, 28, 29] (10/15, 66.67%) mentioned adverse events, but only one trial [28] specifically described these events. Five trials indicated that no adverse events occurred during the whole process. In the trial that reported adverse events, five of the 48 patients in the treatment group had mild nausea, vomiting, and gastrointestinal reactions, but they were able to tolerate the medicine after adding gastric mucosa-protective drugs.

### 3.9. Publication Bias

The number of experiments was insufficient to create a funnel chart, and thus, we cannot judge the publication bias.

## 4. Discussion

Cerebrovascular lesions in CCCI mainly include vasospasm, stenosis, or occlusion of vertebral or internal carotid arteries. The main cause of CCCI in the elderly population in China is atherosclerotic stenosis, especially intracranial stenosis [1, 4, 34]. Both anterior and posterior circulation disorders of the brain can cause CCCI. Anterior circulation includes the internal carotid artery, anterior cerebral artery, and middle cerebral artery. When the function of anterior circulation is disturbed, clinical symptoms such as memory disorder, emotional disorder, or insomnia will appear. Posterior circulation includes the vertebral artery, basilar artery, and posterior cerebral artery. Dizziness, headache, blurred vision, and other clinical symptoms will appear when the posterior circulation is disturbed. Atherosclerotic stenosis causes cerebral artery blood flow velocity. Therefore, measuring the blood flow velocity of anterior and posterior cerebral circulation, including the BA, VA, ICA, middle cerebral artery (MCA), and other arteries, is of great reference value for evaluating the clinical efficacy of drug therapy for CCCI. As a functional reversible ischemic cerebrovascular disease, early detection and intervention of CCCI is of great significance in preventing ischemic stroke, vascular dementia, and other diseases. Western medicine mainly treats different causes and symptoms, such as vascular aspects (atherosclerosis, arteritis, arterial stenosis, etc.), hemodynamics (hypertension and hypotension), blood aspects (hyperlipidemia, diabetes, homocysteine, etc.), and other factors (obesity, smoking, etc.). At present, the main purpose of these treatments is to protect the remaining nerve function and improve and restore the damaged nerve function. In this meta-analysis, we investigated the efficacy of YXQN granules in the treatment of CCCI. The clinical effect of YXQN on cerebral atherosclerosis was observed by comparing the cerebral artery blood flow velocity of the experimental group with that of the control group after treatment. At the same time, the efficacy of TCM symptoms such as headache, dizziness, and insomnia was also examined.

### 4.1. Primary Findings

According to the results of our meta-analysis, the clinical efficacy of YXQN granules in the treatment of CCCI is not negligible. YXQN granules plus routine treatment were superior to nimodipine tablets plus routine treatment in improving TCD results of the BA, VA, and ICA. When comparing the clinical prevalence of headache, dizziness, and insomnia in TCM symptoms, YXQN granules were found to be superior to other drugs, including nimodipine tablets, flunarizine, and troxerutin. TCM is a medical system with a unique theory. As a Chinese patent medicine, YXQN can improve the clinical symptoms of patients. There were differences in the treatment range of the three drugs in the control group. Nimodipine is used for treating aneurysmal subarachnoid hemorrhage and senile cerebral dysfunction [35, 36]. Flunarizine is used for preventing migraine headache and also for treating vertigo caused by vestibular dysfunction [37]. Besides, flunarizine has the potential to preventing or treating the diseases such as cancer, neuropathic pain, diabetes, and Alzheimer's disease because of pharmacological activity of its extensive oxidation resistance and anti-inflammation [38, 39]. In clinic, people use these drugs to alleviate the symptoms caused by chronic cerebral insufficiency but lack targeted treatment and exact curative effect. In this study, we found that the blood flow velocity in the cerebral artery is obviously improved by using YXQN granules than other drugs. If the YXQN granules could change the degree of cerebral artery stenosis, it would supply the reference value of a more in-depth clinical study. Furthermore, the results show that the YXQN granules can alleviate the clinical symptoms of CCCI patients more effectively. The safety of YXQN granules was also examined in this study, and only one experiment reported adverse events.

### 4.2. Limitations

Our study on the efficacy and safety of YXQN granules produced relevant evidence and explanations. However, most of the included studies were published in Chinese journals. There were no specific descriptions of research design, generation of random sequences for randomization, measurement of main results, or allocation blinding. Generally, the quality of the studies was not high, and there may be potential publication and geographical biases. We attempted to contact the research groups to obtain relevant information but obtained little useful feedback. Most reports of adverse events lacked specific descriptions, and further research is needed to prove the safety of the drug. Because of the differences in the outcome indicators of the trials, most of the studies could not be combined for meta-analysis. Because many of the included studies lacked statistical data on the MCA, this study was unable to perform a meta-analysis on the blood flow velocity in the MCA. The effect of YXQN granules on anterior cerebral circulation is still not convincing given our current evidence. TCD of the BA, VA, and ICA was the main outcome indicators; observation of cerebral blood flow can indirectly reflect the curative effect of the medication, but it cannot clearly prove the effect of YXQN granules on CCCI. Although 15 trials were included in this study, the meta-analysis was divided into different subgroups, and funnel charts were not made to identify publication bias and other biases. Advanced and standardized monitoring methods remain necessary in the clinic. Additionally, all the included trials lacked long-term follow-up, which was not comprehensive enough to study the efficacy and safety of YXQN granules.

## 5. Conclusions

Our systematic review and meta-analysis do not allow a definite conclusion on the efficacy and safety of YXQN granules to be drawn, but its role in the clinical treatment of CCCI in TCM cannot be ignored.

## Figures and Tables

**Table 1:** Characteristics of 15 included randomized controlled trials.

ID	Sample size (T/C)	Mean age (years)	Diagnostic standards	Intervention	Comparison	Course duration (days)	Outcome	Adverse effects
Zhou and Liu [24]	86 (46/40)	T: 46 ± 2 C: 48 ± 1.80	Chinese diagnosis and treatment of CCCI guidelines (2004)	YXQN granules 4 g po tid + routine treatment	Flunarizine 5 mg po qn + routine treatment	28	Efficacy rate	None
Ding and Zhang [20]	102 (54/48)	T: 47 C: 49	Chinese diagnosis and treatment of CCCI guidelines (2004)	YXQN granules 4 g po tid + routine treatment	Flunarizine 5 mg po qn + routine treatment	28	Efficacy rate	None
He et al. [21]	65 (34/31)	T: 63.30 ± 5.60 C: 63.70 ± 7.40	Diagnostic criteria from the Japanese Ministry of Health (1990)	YXQN granules 4 g po tid + routine treatment	Nimodipine tablet 20 mg po tid + routine treatment	30	Efficacy rate, hemorheology (erythrocyte, fibrinogen, erythrocyte sedimentation rate)	None
Gu et al. [16]	83 (42/41)	T: 67.20 C: 69.20	Diagnostic criteria from the Japanese Ministry of Health (1990)	YXQN granules 4 g po tid + routine treatment	Nimodipine tablet 20 mg po tid + routine treatment	56	Efficacy rate, TCD (MCA, BA, PCA, VA)	None
Wang [32]	87 (44/43)	T: 62 ± 7.50 C: 61.50 ± 8.50	Diagnostic criteria from the Japanese Ministry of Health (2007)	YXQN granules 4 g po tid + routine treatment	Nimodipine tablet 20 mg po tid + routine treatment	30	Efficacy rate, TCD (ICA, BA, VA)	NR
Chang [25]	52 (26/26)	T: 62.50 ± 4.60 C: 61.30 ± 4.20	Diagnostic criteria from the 16th Stroke Society of Japan (2000)	YXQN granules 4 g po tid + routine treatment	Nimodipine tablet 20 mg po tid + routine treatment	84	Efficacy rate, MMSE, ADL	None
Li [31]	80 (43/37)	T: 62 ± 6.30 C: 63 ± 5.80	Chinese diagnosis and treatment of CCCI guidelines (unclear)	YXQN granules 4 g po tid + routine treatment	Nimodipine tablet 20 mg po tid + routine treatment	30	Efficacy rate, TCD (VA, BA was unclear)	NR
Shen [30]	102 (52/50)	T: 58 ± 6.80 C: 57 ± 7.80	Chinese diagnosis and treatment of CCCI guidelines (2007)	YXQN granules 4 g po tid + routine treatment	Nimodipine tablet 20 mg po tid + routine treatment	30	Efficacy rate, TCD (ICA, BA, VA)	
Wang [33]	74 (37/37)	T: 54.30 ± 3.20 C: 54.20 ± 3.20	Diagnostic criteria from the 16th Stroke Society of Japan (2000)	YXQN granules 4 g po tid + routine treatment	Routine treatment	14	Efficacy rate	NR
Zhu et al. [28]	96 (48/48)	NR	Diagnostic criteria from the 16th Stroke Society of Japan (2000)	YXQN granules 4 g po tid + routine treatment	Routine treatment	14	Different efficacy rate, blood flow	T: 5 C: 0
Li et al. [26]	52 (26/26)	T: 84.60 C: 86.60	Diagnostic criteria for CCCI (unclear)	YXQN granules 4 g po tid + routine treatment	Routine treatment	90	Efficacy rate	None
Liang [27]	54 (27/27)	T: 66.40 C: 62.60	Diagnostic criteria for CCCI (unclear)	YXQN granules 4 g po tid + routine treatment	Troxerutin tablet 0.2 g po tid + routine treatment	90	Efficacy rate	NR
Jia [29]	90 (45/45)	NR	Diagnostic criteria from the 16th Stroke Society of Japan (2000)	YXQN granules 4 g po tid + routine treatment	Troxerutin tablet 0.2 g po tid + routine treatment	90	Efficacy rate, TCD (MCA, PCA, BA)	None
Zhang and Shi [22]	104 (56/48)	T: 56 ± 7 C: 55 ± 5	Diagnostic criteria for CCCI (unclear)	YXQN granules 4 g po tid + routine treatment	Troxerutin tablet 0.2 g po tid + routine treatment	30	Efficacy rate, TCD PI and RI (MCA, ACA, PCA), blood fluid	None
Bi and Chen [23]	84 (42/42)	NR	Chinese diagnosis and treatment of CCCI guidelines (2002)	YXQN granules 4 mg po tid + routine treatment	Cinnarizine tablet 25 mg tid + routine treatment (specific drug was unclear)	30	Efficacy rate, TCD (MCA, VCA, ACA, VA, BA)	None

Routine treatment included antihypertensive, hypoglycemic, and lipid-lowering treatments, or the specific drug was unclear; NR, not reported; T, treatment group; C, control group; TCD transcranial Doppler ultrasound; VA, vertebral artery; BA, basilar artery; MCA, middle cerebral artery; PCA, posterior cerebral artery; ICA, internal carotid artery; ACA, anterior cerebral artery; PI, pulsatility index; RI, resistance index.

**Table 2:** GRADE summary of five randomized controlled trials comparing YXQN granules plus routine treatment with nimodipine tablets plus routine treatment in chronic cerebellar circulation insufficiency.

Outcomes	Anticipated absolute effects^*∗*^ (95% CI)		Relative effect (95% CI)	No. of participants (studies)	Certainty of the evidence (GRADE)
	Assumed risk: nimodipine tablets plus routine treatment	Corresponding risk: YXQN granules plus routine treatment			
BA blood flow velocity	The mean BA blood flow velocity in the control groups was 45.37	The mean BA blood flow velocity in the intervention groups was 3.34-fold higher (2.31- to 4.37-fold higher)	—	272 (3 RCTs)	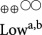
VA blood flow velocity	The mean VA blood flow velocity in the control groups was 40.55	The mean VA blood flow velocity in the intervention groups was 3.21-fold higher (0.62- to 5.8-fold higher)	—	352 (4 RCTs)	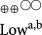
ICA blood flow velocity	The mean ICA blood flow velocity in the control groups was 50.7	The mean ICA blood flow velocity in the intervention groups was 7.46-fold higher (2.01- to 12.9-fold higher)	—	189 (2 RCTs)	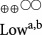
Clinical efficacy of TCM symptoms	642 per 1000	883 per 1,000 (817–927)	RR 4.21 (2.49 to 7.12)	386 (5 RCTs)	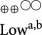

^*∗*^The risk in the intervention group (and its 95% CI) is based on the assumed risk in the comparison group and the relative effect of the intervention (and its 95% CI). CI: confidence interval; RR: risk ratio. GRADE Working Group grades of evidence: high certainty: we are very confident that the true effect lies close to that of the estimate of the effect; moderate certainty: we are moderately confident in the effect estimate. The true effect is likely to be close to the estimate of the effect, but there is a possibility that it is substantially different; low certainty: our confidence in the effect estimate is limited. The true effect may be substantially different from the estimate of the effect; and very low certainty: we have very little confidence in the effect estimate. The true effect is likely to be substantially different from the estimate of the effect. ^a^According to the risk-of-bias graph. ^b^Total number of events was less than 300.

## Supplementary Materials

Supplementary Materials Supplementary materials illustrate the details of risk assessment included in the study, including random sequence generation, allocation concealment, blinding of participants and personnel, blinding of outcome assessment, incomplete outcomes, selective reporting, and other biases. The high risk, low risk, and unclear risk were selected as the results to describe the risks.
