# Supplementary material for: Safety and Clinical Efficacy of Yangxue Qingnao Granules in the Treatment of Chronic Cerebral Circulation Insufficiency: A Systematic Review and Meta-Analysis
Source: Evid Based Complement Alternat Med. 2019 Aug 14;2019:8484263. doi: 10.1155/2019/8484263 (PMC6710749; doi:10.1155/2019/8484263)
Supplement: Supplementary Materials — illustrate the details of risk assessment included in the study, including random sequence generation, allocation concealment, blinding of participants and personnel, blinding of outcome assessment, incomplete outcomes, selective reporting, and other biases. The high risk, low risk, and unclear risk were selected as the results to describe the risks. [file 8484263.f1.pdf]

The risk of bias contents of the included trials

Bi SZ 2007[23]

Authors' judgement / Bias	High risk	Low risk	Unclear risk	Support for judgement
Random sequence generation (selection bias)			√	Unclear based on ”randomized into two groups” and author was not contacted.
Allocation concealment (selection bias)			√	No mention was in the paper and author was not contacted.
Blinding of participants and personnel (performance bias)			√	No specific blind method was described and author was not contacted.
Blinding of outcome assessment (detection bias)	√			We observed through the paper that no blinding on outcomes assessors in the trials.
Incomplete outcome data (attrition)		√		Based on checking the number of patients in Analysis section and randomized number, there might be no missing data.
Selective reporting (reporting bias)		√		Based on checking the outcome measures in Analysis and Method sections, there might be no selective report.
Other bias			√	Unclear due to lack of eligible information.

Chang MJ 2013[25]

Authors' judgement / Bias	High risk	Low risk	Unclear risk	Support for judgement
Random sequence generation (selection bias)			√	Unclear based on ”randomized into two groups”, author was not contacted.
Allocation concealment (selection bias)			√	No mention was in the paper and author was not contacted.
Blinding of participants and personnel (performance bias)			√	No specific blind method was described and author was not contacted.
Blinding of outcome assessment (detection bias)	√			We observed through the paper that no blinding on outcomes assessors in the trials.
Incomplete outcome data (attrition)		√		Based on checking the number of patients in Analysis section and randomized number, there might be no missing data.
Selective reporting (reporting bias)		√		Based on checking the outcome measures in Analysis and Method sections, there might be no selective report.
Other bias			√	Unclear due to lack of eligible information.

Ding XY 2006[20]

Authors' judgement / Bias	High risk	Low risk	Unclear risk	Support for judgement
Random sequence generation (selection bias)			√	Unclear based on ”randomized into two groups”, author was not contacted.
Allocation concealment (selection bias)			√	No mention was in the paper and author was not contacted.
Blinding of participants and personnel (performance bias)	√			We observed through the paper that no blinding was used.
Blinding of outcome assessment (detection bias)	√			We observed through the paper that no blinding on outcomes assessors in the trials.
Incomplete outcome data (attrition)		√		Based on checking the number of patients in Analysis section and randomized number, there might be no missing data.
Selective reporting (reporting bias)	√			Adverse events were not described in detail, there might be selective reporting.
Other bias			√	Unclear due to lack of eligible information.

Gu XX 2005[16]

Authors' judgement / Bias	High risk	Low risk	Unclear risk	Support for judgement
Random sequence generation (selection bias)			√	Unclear based on ”randomized into two groups”, author was not contacted.
Allocation concealment (selection bias)			√	No mention was in the paper and author was not contacted.
Blinding of participants and personnel (performance bias)	√			We observed through the paper that no blinding was used.
Blinding of outcome assessment (detection bias)	√			We observed through the paper that no blinding on outcomes assessors in the trials.
Incomplete outcome data (attrition)		√		Based on checking the number of patients in Analysis section and randomized number, there might be no missing data.
Selective reporting (reporting bias)		√		Based on checking the outcome measures in Analysis and Method sections, there might be no selective report.
Other bias			√	Unclear due to lack of eligible information.

He P 2013[21]

Authors' judgement / Bias	High risk	Low risk	Unclear risk	Support for judgement
Random sequence generation (selection bias)			√	Unclear based on "randomized into two groups", author was not contacted.
Allocation concealment (selection bias)			√	No mention was in the paper and author was not contacted.
Blinding of participants and personnel (performance bias)	√			We observed through the paper that no blinding was used.
Blinding of outcome assessment (detection bias)	√			We observed through the paper that no blinding on outcomes assessors in the trials.
Incomplete outcome data (attrition)		√		Based on checking the number of patients in Analysis section and randomized number, there might be no missing data.
Selective reporting (reporting bias)	√			Adverse events were not described in detail, there might be selective reporting.
Other bias			√	Unclear due to lack of eligible information.

Jia ZC 2011[29]

Authors' judgement / Bias	High risk	Low risk	Unclear risk	Support for judgement
Random sequence generation (selection bias)			√	Unclear based on "randomized into two groups", author was not contacted.
Allocation concealment (selection bias)			√	No mention was in the paper and author was not contacted.
Blinding of participants and personnel (performance bias)	√			We observed through the paper that no blinding was used.
Blinding of outcome assessment (detection bias)	√			We observed through the paper that no blinding on outcomes assessors in the trials.
Incomplete outcome data (attrition)		√		Based on checking the number of patients in Analysis section and randomized number, there might be no missing data.
Selective reporting (reporting bias)	√			Adverse events were not described in detail, there might be selective reporting.
Other bias			√	Unclear due to lack of eligible information.

Li DR 2003[31]

Bias \ Authors' judgement	High risk	Low risk	Unclear risk	Support for judgement
Random sequence generation (selection bias)		√		We observed through the paper that random number table grouping was used.
Allocation concealment (selection bias)			√	No mention was in the paper and author was not contacted.
Blinding of participants and personnel (performance bias)		√		We observed the use of single-blind method through the paper.
Blinding of outcome assessment (detection bias)	√			We observed through the paper that no blinding on outcomes assessors in the trials.
Incomplete outcome data (attrition)		√		Based on checking the number of patients in Analysis section and randomized number, there might be no missing data.
Selective reporting (reporting bias)		√		Based on checking the outcome measures in Analysis and Method sections, there might be no selective report.
Other bias			√	Unclear due to lack of eligible information.

Li JK 2013[26]

Bias \ Authors' judgement	High risk	Low risk	Unclear risk	Support for judgement
Random sequence generation (selection bias)			√	Unclear based on "randomized into two groups", author was not contacted.
Allocation concealment (selection bias)			√	No mention was in the paper and author was not contacted.
Blinding of participants and personnel (performance bias)	√			We observed through the paper that no blinding was used.
Blinding of outcome assessment (detection bias)	√			We observed through the paper that no blinding on outcomes assessors in the trials.
Incomplete outcome data (attrition)		√		Based on checking the number of patients in Analysis section and randomized number, there might be no missing data.
Selective reporting (reporting bias)	√			Adverse events were not described in detail, there might be selective reporting.
Other bias			√	Unclear due to lack of eligible information.

Liang Y 2012[27]

Authors' judgement / Bias	High risk	Low risk	Unclear risk	Support for judgement
Random sequence generation (selection bias)			√	Unclear based on "randomized into two groups", author was not contacted.
Allocation concealment (selection bias)			√	No mention was in the paper and author was not contacted.
Blinding of participants and personnel (performance bias)	√			We observed through the paper that no blinding was used.
Blinding of outcome assessment (detection bias)	√			We observed through the paper that no blinding on outcomes assessors in the trials.
Incomplete outcome data (attrition)		√		Based on checking the number of patients in Analysis section and randomized number, there might be no missing data.
Selective reporting (reporting bias)	√			Adverse events were not described in detail, there might be selective reporting.
Other bias			√	Unclear due to lack of eligible information.

Shen HJ 2010[30]

Authors' judgement / Bias	High risk	Low risk	Unclear risk	Support for judgement
Random sequence generation (selection bias)			√	Unclear based on "randomized into two groups", author was not contacted.
Allocation concealment (selection bias)			√	No mention was in the paper and author was not contacted.
Blinding of participants and personnel (performance bias)	√			We observed through the paper that no blinding was used.
Blinding of outcome assessment (detection bias)	√			We observed through the paper that no blinding on outcomes assessors in the trials.
Incomplete outcome data (attrition)		√		Based on checking the number of patients in Analysis section and randomized number, there might be no missing data.
Selective reporting (reporting bias)	√			Adverse events were not described in detail, there might be selective reporting.
Other bias			√	Unclear due to lack of eligible information.

Wang SJ 2017[33]

Authors' judgement / Bias	High risk	Low risk	Unclear risk	Support for judgement
Random sequence generation (selection bias)			√	Unclear based on "randomized into two groups", author was not contacted.
Allocation concealment (selection bias)			√	No mention was in the paper and author was not contacted.
Blinding of participants and personnel (performance bias)	√			We observed through the paper that no blinding was used.
Blinding of outcome assessment (detection bias)	√			We observed through the paper that no blinding on outcomes assessors in the trials.
Incomplete outcome data (attrition)		√		Based on checking the number of patients in Analysis section and randomized number, there might be no missing data.
Selective reporting (reporting bias)	√			Adverse events were not described in detail, there might be selective reporting.
Other bias			√	Unclear due to lack of eligible information.

WangWP 2010[32]

Authors' judgement / Bias	High risk	Low risk	Unclear risk	Support for judgement
Random sequence generation (selection bias)			√	Unclear based on "randomized into two groups", author was not contacted.
Allocation concealment (selection bias)			√	No mention was in the paper and author was not contacted.
Blinding of participants and personnel (performance bias)			√	No specific blind method was described and author was not contacted.
Blinding of outcome assessment (detection bias)	√			We observed through the paper that no blinding on outcomes assessors in the trials.
Incomplete outcome data (attrition)		√		Based on checking the number of patients in Analysis section and randomized number, there might be no missing data.
Selective reporting (reporting bias)	√			Adverse events were not described in detail, there might be selective reporting.
Other bias			√	Unclear due to lack of eligible information.

Zhang AM 2008[22]

Authors' judgement / Bias	High risk	Low risk	Unclear risk	Support for judgement
Random sequence generation (selection bias)			√	Unclear based on "randomized into two groups", author was not contacted.
Allocation concealment (selection bias)			√	No mention was in the paper and author was not contacted.
Blinding of participants and personnel (performance bias)	√			We observed through the paper that no blinding was used.
Blinding of outcome assessment (detection bias)	√			We observed through the paper that no blinding on outcomes assessors in the trials.
Incomplete outcome data (attrition)		√		Based on checking the number of patients in Analysis section and randomized number, there might be no missing data.
Selective reporting (reporting bias)	√			Adverse events were not described in detail, there might be selective reporting.
Other bias			√	Unclear due to lack of eligible information.

Zhou JM 2008[24]

Authors' judgement / Bias	High risk	Low risk	Unclear risk	Support for judgement
Random sequence generation (selection bias)			√	Unclear based on "randomized into two groups", author was not contacted.
Allocation concealment (selection bias)			√	No mention was in the paper and author was not contacted.
Blinding of participants and personnel (performance bias)	√			We observed through the paper that no blinding was used.
Blinding of outcome assessment (detection bias)	√			We observed through the paper that no blinding on outcomes assessors in the trials.
Incomplete outcome data (attrition)		√		Based on checking the number of patients in Analysis section and randomized number, there might be no missing data.
Selective reporting (reporting bias)		√		Based on checking the outcome measures in Analysis and Method sections, there might be no selective report.
Other bias			√	Unclear due to lack of eligible information.

Zhu Y 2011[28]

Bias \ Authors' judgement	High risk	Low risk	Unclear risk	Support for judgement
Random sequence generation (selection bias)			√	Unclear based on "randomized into two groups", author was not contacted.
Allocation concealment (selection bias)			√	No mention was in the paper and author was not contacted.
Blinding of participants and personnel (performance bias)			√	No specific blind method was described and author was not contacted.
Blinding of outcome assessment (detection bias)	√			We observed through the paper that no blinding on outcomes assessors in the trials.
Incomplete outcome data (attrition)		√		Based on checking the number of patients in Analysis section and randomized number, there might be no missing data.
Selective reporting (reporting bias)		√		Based on checking the outcome measures in Analysis and Method sections, there might be no selective report.
Other bias			√	Unclear due to lack of eligible information.
